# Who wants ‘the worst of the worst’? Rationales for and consequences of third country resettlement of Guantanamo Bay detainees

**DOI:** 10.1007/s10611-020-09932-z

**Published:** 2021-04-07

**Authors:** Gaia Rietveld, Joris van Wijk, Maarten P. Bolhuis

**Affiliations:** 1 Leiden University, Leiden, Netherlands; 2 Vrije Universiteit Amsterdam, Amsterdam, Netherlands

## Abstract

Against the backdrop of countries increasingly being confronted with undesirable but unreturnable non-citizen terrorist suspects, this article describes the resettlement process of 150 cleared but unreturnable Guantanamo Bay detainees. Merely 13% of these detainees have been resettled in full democracies, compared to 52% in authoritarian regimes. Using Starkley et al.’s concept of ‘zone agreement’ the article explains how the U.S. particularly managed to incentivize pragmatically oriented – rather than idealistically motivated – governments to engage in third country resettlement [16]. From the perspective of the U.S. the resettlement scheme can be considered relatively successful, while the experiences of resettlement countries and the resettled detainees themselves have been very mixed.

## Introduction

In recent years there has been increased attention for the ‘securitisation of migration’ (the presentation of migration as a security problem; [1]) and ‘crimmigration’ (the merger of criminal law and immigration law; [2]). The rise of transnational terrorism, the foreign fighter phenomenon and fears of ‘Jihadist infiltration in migration flows’ [3] have led to ever more rigid counterterrorism approaches [4] where we can observe a merger between national security law and immigration law. Alleged or convicted terrorist citizens are increasingly stripped of their citizenship, while alleged or convicted non-citizens are increasingly denied access or residence permits. Consequently, states are ever more confronted with undesirable non-citizens who are considered to pose a threat to national security, but cannot be deported to their country of origin.

Turkey, Syria, Iraq and the Kurdish authorities in Northern Syria currently detain hundreds of undeportable non-citizens suspected to have been fighting for Islamic State [5], while countries like Australia, Canada, France, India, the Netherlands and the United Kingdom have over the past years struggled how to treat suspected or convicted foreign terrorists who cannot be deported [6]. The limbo-situation of these ‘undesirable but unreturnable’ migrants (UBUs) results from a range of factors, including non-cooperation on behalf of the State of origin, a lack of transport options to enforce removal or international human rights impediments blocking *refoulement* [7].

Worldwide, countries have developed strategies in dealing with UBUs, ranging from strictly monitoring their whereabouts and keeping them in long-term detention, to requesting diplomatic assurances that they will not be subjected to mistreatment upon return. Many of these approaches have been critiqued for being ad hoc and inconsistent character, unregulated, overly restrictive and failing “to provide a sensible or sustainable solution to either the legal and political concerns of the State or the anomalous situation of the individual involved” [6]. Another, less often applied, policy response in governments’ ‘toolbox’ to deal with UBUs is their resettlement to third countries. This, in principle, could offer a sustainable solution for the State as well as the UBU. This article discusses the resettlement process of arguably *the* most hotly debated UBUs in history: cleared and unreturnable Guantánamo detainees.

Since its opening roughly 780 men have been detained in Guantánamo Bay, originating from over 35 countries [8]. In justifying the existence and necessity of the detention camp, top Department of Defense (DoD) officials have regularly claimed Guantánamo Bay is reserved for the ‘worst of the worst’.^1^ However, the varying Guantánamo detainee review mechanisms that have existed over the years,^2^ actually determined the majority of detainees to be low-level offenders – if offenders at all – who were not affiliated with organizations on the Department of Home Security terrorist watchlists [11, 14]. As a result, many detainees have since 2002 been cleared for release or cleared for transfer,^3^ with only 40 of the overall 780 detainees remaining at Guantánamo Bay by May 2020, the time of writing [8]. While the majority of cleared detainees have been transferred back to their country of origin, a significant number of detainees cleared for release have been considered unfit to return to their home country. As a result, according to the New York Times Guantánamo Docket database [8], the U.S. government has been ‘stuck’ with 172 *cleared, but unreturnable Guantánamo detainees* which have over the years been transferred to a large variety of third countries.

While third country resettlements may have been the most viable – if not the only – alternative to indefinite detention, they are challenging to realize. Third countries are in no way obligated to take in Guantánamo detainees, and therefore the U.S. is fully dependent on their willingness to do so. On the face of it, there are no clear direct benefits that would make resettling Guantánamo detainees appealing to these countries. Somehow, however, the U.S. has succeeded in doing so.

In light of the wider discussion on the merger between national security and migration law and the question how states can deal, should deal or do deal with UBUs, this article offers an empirical analysis on the resettlement process of unreturnable Guantanamo Bay detainees. More specifically, it aims to: 1) provide an empirical overview of states that resettled cleared and unreturnable Guantanamo Bay detainees; 2) explain why some states resettled these UBUs while others have not; and 3) discuss to what extent, from the perspective of U.S., the resettlement countries and the resettled detainees themselves, the resettlement process can be considered a successful ‘way out of limbo’ or not. A systematic analysis of the different scenarios that unfold when states seek the cooperation of other states to resettle UBUs enhances the understanding of both academics and policymakers of the dynamics in such situations. These insights can be extended to situations beyond Guantanamo, as many states are now or will most likely in the near future be confronted with an increasing number of UBUs associated with terrorism.

After a brief methodological paragraph, section three provides a more detailed outline of the reasons why the U.S. considers certain detainees unreturnable to their country of origin. The fourth section reviews which countries have been destinations for resettlement and why. Using Starkley et al. ‘zone of agreement’ concept it analyzes how the U.S. has gone about finding countries willing to resettle detainees, and what has possibly motivated countries to host them [16]. Section five examines the consequences of the resettlement process, differentiating the effects for the U.S. and the resettlement countries, as well as for the resettled individuals concerned. Ultimately, section six concludes that the U.S. particularly managed to incentivize pragmatically oriented – rather than idealistically motivated – governments to engage in third country resettlement. From the perspective of the U.S. the resettlement scheme can be considered relatively successful, but the experiences of resettlement countries and the resettled detainees themselves have been very mixed.

## Methodology

This explorative study is based on an extensive literature review consisting of an analysis of relevant academic scholarship, policy documents of governmental, non-governmental and intergovernmental organizations, as well as publications in (online) popular media. Particularly useful in answering the second research question were leaked U.S. diplomatic cables made available by Wikileaks. Similar to other research methods that are predominantly deployed in journalism but also have potential for academics,^4^ these cables offered valuable information that would otherwise have been very difficult to obtain.

In assessing the whereabouts of cleared but unreturnable Guantánamo Bay detainees we used The New York Times Guantanamo Docket Database as a starting point for our analysis. By May 2020 the database listed 172 detainees as ‘transferred to a third country’. Based on publicly available information we carefully reviewed all these cases to assess whether they met both of the following criteria:

- The detainee is transferred to a third country:
  - of which he had not possessed nationality and/or of which he has not been a legal resident, prior to detention at Guantánamo, and
  - with the objective to allow the ex-detainee to temporarily or permanently reside in the resettlement country.^5^

This review led us to exclude 22 detainees from the Docket Database list, leaving us with a total of 150 ‘resettled’ detainees. An annex detailing which detainees have been excluded from our database of cases, and why, is presented at the end of the article. Reasons to conclude that individuals did not meet one or both of the criteria were diverse, but for example included the fact that it turned out they held dual nationality (including the nationality of the ‘resettlement country’), that they had legally resided in the resettlement country prior to detention or that they were essentially extradited, rather than transferred for the purpose of temporary or permanent resettlement.

## The issue of unreturnability

There are three reasons for the U.S. to label certain detainees as unable to return to their country of origin, in whose cases there is a need for third country resettlement as transfers to U.S. soil are prohibited. *First*, some detainees are deemed unreturnable because of human rights concerns. As a signatory to the UN Convention against Torture and Other Cruel, Inhumane, or Degrading Treatment or Punishment (UNCAT), the U.S. cannot ‘expel, return (“*refouler*”) or extradite a person to another State where there are substantial grounds for believing that he would be in danger of being subjected to torture’ in conformity with art. 3(1).^6^ In its Final Report the Guantanamo Review Task Force [19] upholds that U.S. policy in arranging Guantánamo Bay transfers is motivated by these UNCAT provisions. It furthermore asserts that in certain cases detainees with a well-founded fear of prosecution are entitled to protection against persecution, in conformity with the 1967 Protocol Relating to the Status of Refugees (ibid.). This stance was corroborated by former Special Envoy for Guantanamo Closure Lee S. Wolosky, stating in an address to the House Foreign Affairs Committee that all transfers are conducted in a manner that ‘is consistent with our long-standing policy on humane treatment’ [20]. Thus, if the Department of State assesses fears of torture and/or prosecution upon return to be grounded, transfer to the country of origin is deemed impossible [21].

While generally this assessment is made individually for every detainee [20], there is a group of Guantánamo detainees that as a whole have been assessed as unreturnable due to human rights concerns: Chinese detainees of Uyghur ethnicity. They therefore function as a good illustration of such cases. The Uyghur are a Muslim ethnic minority native to the western Xinjiang region in China.^7^ Since 2002, 22 Uyghurs have been detained in Guantánamo as suspected members of the East Turkistan Islamic Movement (ETIM). The ETIM is an Uyghur separatist movement designated as a terrorist organization in 2002 by e.g. the UN Security Council and the U.S. after lobbying by the Chinese government [24, 25].^8^ However, the Uyghur Guantánamo cases were shrouded with uncertainty due to a lack of evidence regarding the detainees’ involvement in any anti-U.S. terrorism activity [28], and as of 2008 all Uyghur detainees are no longer classified as enemy combatants.^9^ As China considers all of the Guantanamo Uyghurs to be terrorist suspects it has urged for their return to face justice domestically. However, the U.S. has refused to extradite or repatriate them to China, listing fears of maltreatment upon return as the reason for its decision to resettle them instead [29–31]. Apart from Chinese Uyghurs, it is on the basis of publicly available documentation not possible to provide a complete overview of all individuals or groups of nationals who have been unreturnable to their country of origin because of human rights concerns.

A *second* reason for the U.S. to refrain from transferring cleared detainees to their country of origin are security concerns. Transfers of detainees are only possible if the resettlement country can give sufficient assurances that it is able and willing to mitigate the risk of the detainee in question from posing a security threat to the U.S. [20, 32]. Thus, detainees are considered unreturnable if their country of origin is regarded to be unwilling and/or unable to offer these assurances. Again, the question when security concerns warrant a third country resettlement comes down to individual case assessments. However, some countries are considered to have such an unstable security environment that there is a general prohibition on transferring any detainees to that country. Any cleared detainees originating from these countries thereby automatically require a third country resettlement. For instance, through NDAA legislation Libya, Somalia and Syria have been prohibited as transfer destinations since 2016,^10^ while in 2017 Yemen was added to this list.^11^ Furthermore, there had already been a moratorium on transfers to Yemen from December 2009, after an attempted bombing of a plane headed for Detroit by an Al Qaeda branch based in Yemen, although this moratorium was partially revoked in May 2013 ([19, 30, 31], p. 18). As Yemenites are – after Afghans and Saudi Arabians – in number the third nationality that has been detained in Guantánamo Bay with a total of 115 detainees, the moratorium and prohibition on transfers, together with the overall unstable security environment of Yemen, have proven a significant challenge for removing cleared Yemenite detainees out of the Guantánamo facility. As a result, there have been no transfers to Yemen since July 2010, and at the time of writing 76 Yemenites have been resettled to a variety of third countries [8]. Apart from the abovementioned countries where there has been a general prohibition on transferring any detainees, it is again – because such information is not publicly available – not possible to give a complete overview of individuals or nationalities that have not been returned to their country of origin because of security concerns.

*Finally*, there are those detainees that are stateless, and therefore do not have a country of origin to be returned to. Currently, the only (resettled) detainees that fall into this category originate from the Palestinian territories. Many states including the U.S. do not recognize passports issued by Palestinian authorities as a proof of legal citizenship, thereby rendering inhabitants of the Palestinian territories *de jure* stateless [33]. As a result, detainees of Palestinian origin cannot be returned. Five stateless detainees of Palestinian origin have been held at the Guantánamo facilities, four of whom have so far been resettled [8]. The last Palestinian remains in Guantánamo without charges, as the Periodic Review Board considers continued detention warranted for security purposes [34].

The issue of cleared but unreturnable detainees became especially pressing under the Obama administration. On his third day in office, 22 January 2009, Obama signed an executive order requiring the closure of Guantánamo Bay and the immediate review of every Guantánamo detainee.^12^ His vow to empty the facility created the need to properly address the matter of cleared but unreturnable detainees, many of whom remained detained at Guantánamo Bay. While the 240 detainees remaining at the detention camp in January 2009 were reviewed for potential release throughout the year, the closure of Guantánamo Bay was met with broad opposition in Congress [35]. As a result, the National Defense Authorization Act (NDAA) for fiscal year 2010, passed by Congress in October 2009, included provisions prohibiting the use of government funds for releasing Guantánamo detainees to U.S. soil.^13^ Eventually the NDAA for fiscal year 2011, passed in January 2011, fully prohibited any transfers as well as releases to U.S. soil.^14^ As it was impossible to release or transfer cleared detainees to either their country of origin or the U.S., the Obama administration adopted a method which the preceding Bush-government had already tentatively used before: resettling cleared detainees to third countries willing to take them in.

Very little is publicly known about the evaluation process for deciding whether human rights concerns or security concerns necessitate third country resettlement of a cleared detainee. Under the Obama administration the Office of the Special Envoy for Guantánamo Closure was set up to carry out these evaluations as part of its responsibility for all diplomatic issues related to the Guantanamo Bay facility, including transfer negotiations and post-transfer monitoring [20, 36]. However, as the Office of the Special Envoy for Guantanamo Closure was dismantled in 2017 under the Trump administration [37], it is unclear which government institutions currently oversee detainee evaluation and transfer negotiations.

## Patterns and trends in third country resettlements

The first of the 150 detainees to be resettled were five Chinese Uyghur men that were transferred to Albania in May 2006 [8]. Since then ex-detainees have either temporarily or permanently been resettled to 29 different countries all over the world.^15^ This section gives more insight into the patterns and trends of the 150 effectuated third country resettlements.

**Fig. 1:**
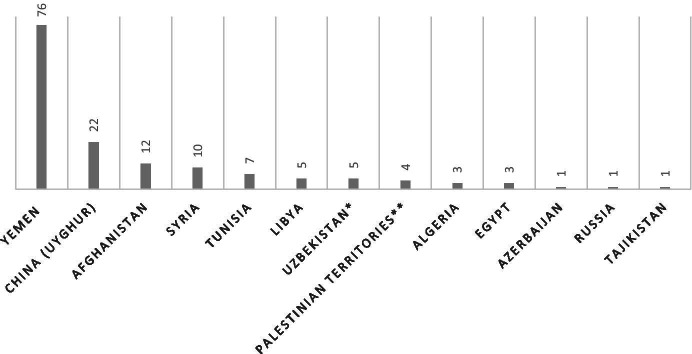
Number of resettled ex Guantánamo Bay-detainees per country of origin 2002-2020. * One Uzbeki ex-detainee possesses dual nationality, Russian being his second nationality. ** One Palestinian ex-detainee possesses dual nationality, Saudi Arabian being his second nationality

As demonstrated in Fig. 1, the vast majority (76) of resettled ex-detainees are Yemenites, followed by Chinese Uyghurs (22) and Afghans (12). As discussed in the previous paragraph, Yemen has long been considered unfit to return detainees to, while Yemenites form the third largest nationality that has been detained at Guantánamo. This explains the large proportion of Yemenites among the resettled detainees. The high number of Chinese Uyghurs follows the aforementioned unwillingness of the U.S. to return any of them to China’s custody over post-release treatment concerns. As a result, all detained Chinese Uyghurs, without exception, have been resettled. While the unstable security situation and flawed human rights track record in Afghanistan over the past two decades could seemingly explain their position as third, it is important to note that most of the 220 Afghan detainees in Guantánamo Bay have actually been returned to their home country. Of the merely twelve who were resettled, five detainees were resettled in a highly exceptional context. They were all former high-ranking members of the Taliban who were sent to Qatar as part of a deal with the Taliban in order to free Bowe Bergdahl, an American soldier that was held captive for years by the Taliban [40]. While none of these detainees had faced any charges, it is highly unlikely the U.S. would have cleared them for release – and hence, resettled – if not for the Bergdahl deal [41]. Nonetheless, security concerns did seem to play a role in these negotiations, as reportedly the Qatar’s guarantee to monitor the detainees was an important factor in convincing all relevant U.S. parties all risks would be mitigated [42]. As for the remaining seven detainees, there is no public information that discloses why the U.S. assessed there were security or human rights concerns that necessitated third country resettlement in these specific cases.

**Fig. 2:**
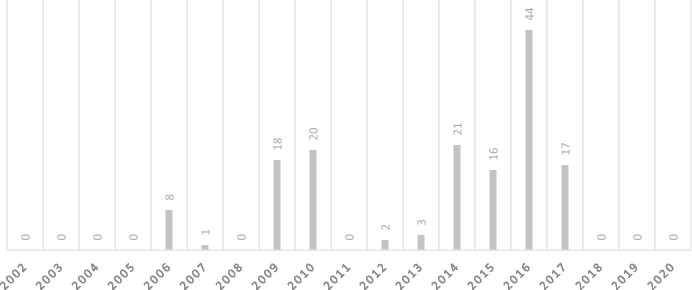
Number of third country resettlements of ex Guantánamo Bay-detainees 2002-2020

As Fig. 2 shows, all third country resettlements took place between 2006 and 2017. The pattern in third country resettlements over time strongly reflects the changes in administrations that since the opening of Guantánamo Bay. From its opening in 2002 until January 20, 2009 the U.S. was under the authority of the Republican Bush administration. While during this time some third country resettlements did take place, this makes up only 6% of all resettlements. The remaining 94% of resettlements took place under the Democratic Obama administration, which was in power from January 20, 2009 until January 20, 2017. This reflects the Obama administration’s commitment to close the Guantánamo Bay facility. Especially noteworthy is the enormous spike in resettlements towards the end of the Obama administration in 2016 and 2017, which accounts for 41% of all third country resettlements. Most likely this spike can be explained by an attempt to effectuate as many third country resettlements as possible before the next Republican administration under Donald Trump would come to power. Throughout his presidential campaign, Trump had been quite vocal that closure of Guantanamo Bay would under his presidency not be a priority. During a 2016 campaign rally in Indiana he rather said: “We're not closing Gitmo, we're going to fill it up!” [43]. All resettlements in 2017 were effectuated before the inauguration of President Trump on January 20, 2017, with the last three resettlements taking place on the 19^th^ of January, the very last day Obama was in office. Since then no more detainees have been resettled to a third country.

Resettlement countries: geographical spread

**Fig. 3:**
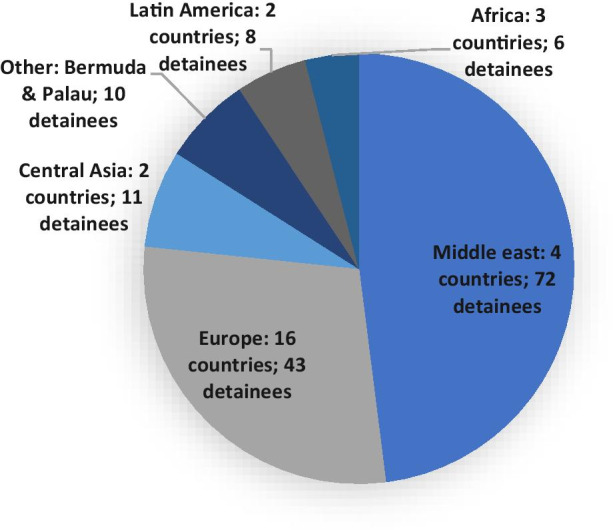
Number of resettled ex-detainees per region

**Fig. 4:**
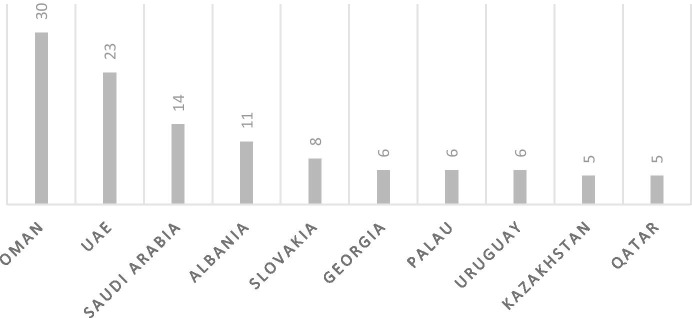
Top 10 resettlement countries by number of resettled ex-detainees

As mentioned above, 29 countries have so far taken in ex-detainees with foreign nationalities. Looking at the geographical spread of these countries as presented in Fig. 3, it becomes clear that while both Europe and the Middle East have been important resettlement destinations, European countries tend to take in way less detainees each than Middle Eastern countries. From Fig. 4 it can be deduced that the 10 countries that have taken in most ex-detainees together account for 76% of all resettled ex-detainees, which means that the remaining 19 countries have each resettled only a limited number of detainees (four or less). The three countries that have taken in the most detainees are Oman, the United Arab Emirates (UAE) and Saudi Arabia, accounting for 45% of all third country resettlements. Virtually all these resettlements took place towards the end of the Obama administration in 2015, 2016 and 2017.

Arguably, traditional U.S. allies would be most prepared to help out the U.S. and take in detainees. However, some important U.S. allies are notably missing, such as Canada, Australia and even the United Kingdom. As a matter of fact, it is striking to note that merely 23% of the unreturnable detainees has been resettled to NATO allies. This implies that shared political values and good diplomatic and military relations between the U.S. and a third country alone cannot explain why a country is prepared to resettle one or more detainees. That said, though they may not be members of NATO, Oman, the UAE and Saudi Arabia can certainly all be considered ‘friends’ of the U.S government. All are major U.S. arms importers and have a longstanding and intensive military cooperation with the U.S., while all have also consistently supported U.S.’s ‘war on terror’.^16^ As the NATO countries, Oman, the UAE and Saudi Arabia together account for 68% of all resettled detainees, it seems that while established relations cannot fully explain preparedness for resettlement, it can be an important contributing factor.

Resettlement countries: characteristics

**Fig. 5:**
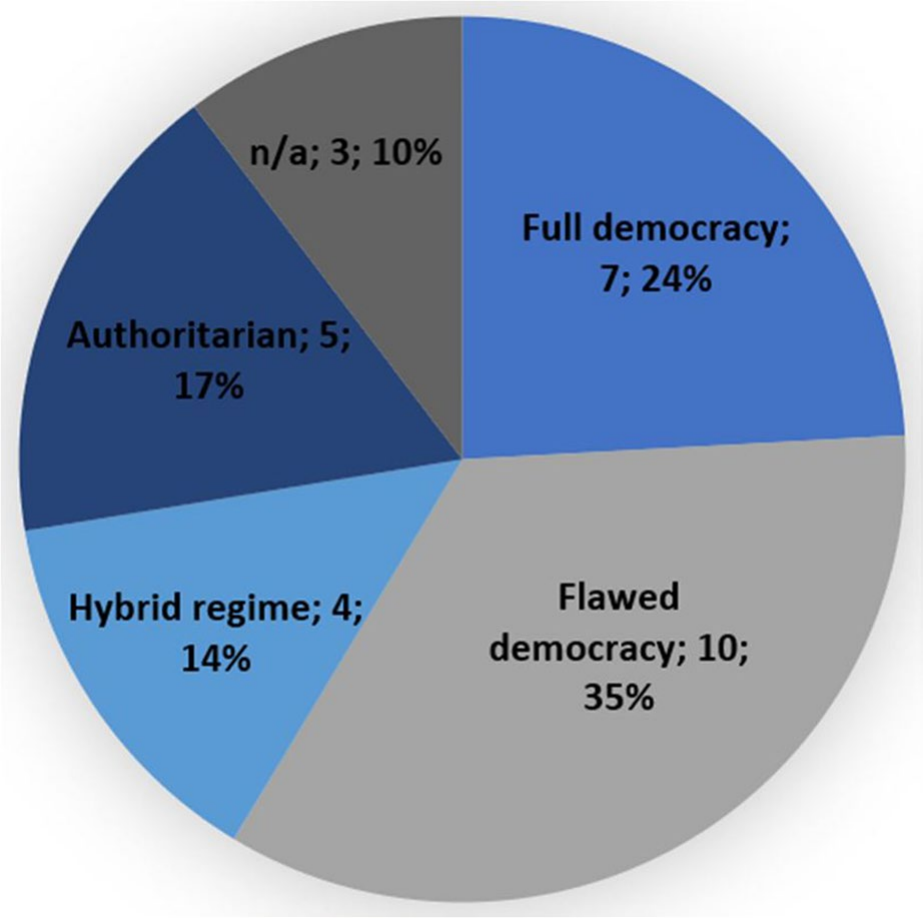
Number of countries per regime type

**Fig. 6:**
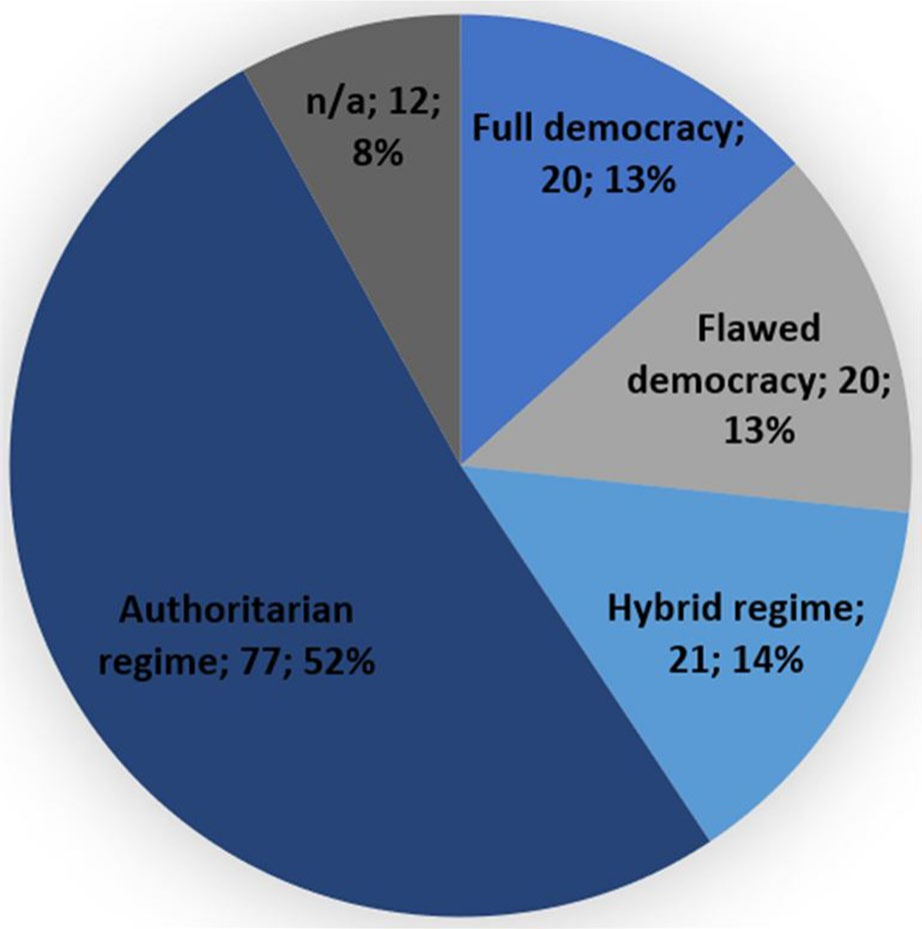
Number of detainees per regime type

Using the Economist Democracy Index 2019 for reference,^17^ Figs. 5 and 6 demonstrate the regime types that detainees have been resettled to. Fig. 5 shows that of all the 29 resettlement countries only roughly a quarter (24%)^18^ can be considered a ‘full democracy’. The remaining countries can be defined as flawed democracies (35%),^19^ hybrid regimes (14%),^20^ or authoritarian regimes (17%).^21^ In addition to this, Fig. 6 demonstrates that 52% of the resettled detainees ended up in a third country under an authoritarian regime, 14% under a hybrid regime, 13% under a flawed democracy and only 13% under a full democracy.^22^ Indeed, authoritarian regimes have taken in a disproportionately high number of detainees, while the two categories of democratic regimes – even when taken together – account for a disproportionately low number of resettled detainees. This indicates there might not only be a connection between the regime type and the willingness to resettle detainees, but also between the regime type and the number of detainees a country is willing to take in. This hypothesis is supported by the fact that the top 3 resettlement countries, as listed in Fig. 4, are all categorized as authoritarian regimes. Many of these authoritarian regimes score low on human rights indexes. To illustrate, Oman, the UAE and Saudi Arabia were each ranked 134^th^, 128^th^ and 149^th^ respectively out of 162 countries in the Human Freedom Index 2019, while fully democratic resettlement countries that have taken in far fewer detainees such as Germany, Ireland and Switzerland each ranked 13^th^, 8^th^ and 2^nd^ respectively [49]. To understand why detainees have been taken in by so many different countries with such different regime types and ties to the U.S., one must take a closer look at the negotiation process that precedes resettlements, as will be done in the next section.

## Negotiating resettlements

The ‘UBU-phenomenon’ is not new and neither is the strategy to promote the resettlement of undeportable non-citizens who are believed to pose a threat to national security to a third country. Already from the 1920s well into the 1950s the United States (U.S.) and Canada were faced with the dilemma how to deal with Russian anarchist ‘terrorists’ or communist ‘radicals’ who could not be deported [50–52]. In 1950, as an alternative to indefinite detention, the U.S. House of Representatives already discussed the possibility to transfer undeportable communist ‘subversives’ to “any country which will agree to accept such aliens in its territory.” Congressman Cellar had strong reservations, arguing that the combination of the Attorney General’s right to designate a place for detention and third country transfers could result in – what we would now refer to as – realpolitikal powerplay to ‘outsource’ the UBU-problem:

‘The Attorney General (…) may have them detained outside the country. He may induce Great Britain, for example, to set up a place of detention or a stockade on the island of Mauritius in the Indian Ocean. He may persuade the French Government, under penalty of having Marshall plan aid cut off – and that is, of course, an extreme case – to set up a Devil’s Island off French Guinea, where these persons may be shipped’ (U.S. Congressional Record 1950: 10450).

Cellar’s reservations were set aside and by 1952 the McCarran-Walter Act empowered the U.S. Attorney General to deport otherwise unremovable aliens to any country willing to accept them. At the time, it however did not solve U.S. government’s problem in dealing with unreturnable subversives. Negotiating resettlements proved more challenging than anticipated. In 1956, four years after the act was passed, Maslow commented dryly that “those countries to which the alien has no previous ties usually decline to accept him as a deportee” ([51]: 319). How can we explain that more than 50 years later, a surprisingly high number of ‘countries to which the alien has no previous ties’ decided to resettle Guantanamo Bay detainees?

**Fig. 7:**
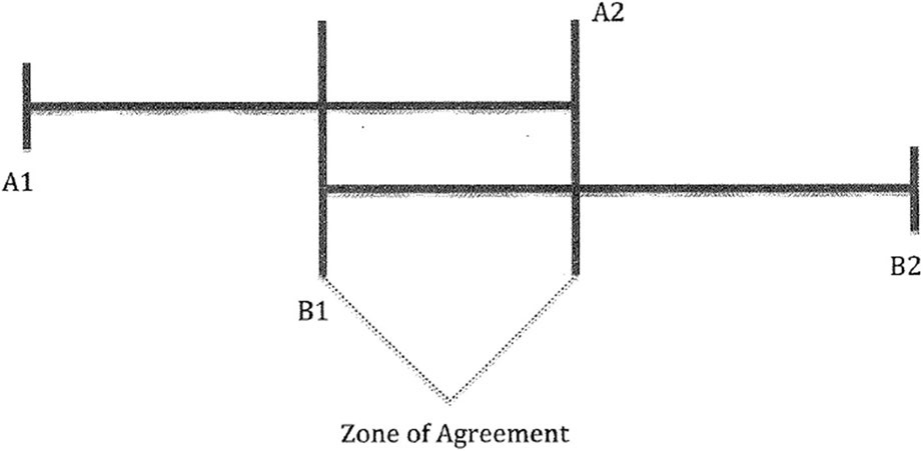
The zone of agreement ([16], 130)

All third country resettlements of Guantanamo Bay detainees are made possible through an intensive negotiation process between the U.S. and the resettlement country. As mentioned in the introduction resettlement countries are in no way obligated to take in detainees, nor are there seemingly clear direct benefits in doing so. Therefore, closer examination of the negotiation process is needed to understand why third countries end up agreeing to take in detainees. Starkley et al. provide a useful concept to understand what strategic approaches are employed during international negotiation processes: the ‘zone of agreement’ [16]. As visualized in Fig. 7, each party (A and B respectively) will at the start of a negotiation have a minimum position, represented by A1 and B2. During negotiations each party will have to do concessions from their minimum position in order to approximate a situation to which both parties can agree. However, parties are only willing or able to go so far when they do concessions, represented by B1 and A2 in Fig. 7. What lies in between these two extremes is the ‘zone of agreement’ in which agreements can be made. Negotiations can thus only result in agreement when the extremes of concession cross each other to create a zone of agreement (ibid., 129-131).

During negotiations incentives can be used by one party to convince the other to do a concession towards the zone of agreement. These incentives can take on a competitive or collaborative character. When there are common interests between both parties, collaborative negotiation is possible, also called the ‘carrot approach’. Here positive incentives in the interest of the other party are used to come to an agreement. However, when positive incentives cannot induce agreement, parties can turn to competitive negotiation, also called the ‘stick approach’. In that case incentives of a more coercive nature are employed to compel the other party into agreement. Which strategy is most effective is mostly dependent on the relationship between the negotiating parties and the willingness and ability of negotiation parties to do concessions, which can be influenced by outside factors as well (ibid., 131-140).

While very little is publicly known regarding the specifics of Guantanamo detainee resettlement negotiation processes, we can understand the dynamics of these processes by applying the concept of the zone of agreement. In the Guantanamo case the U.S. is the negotiating party that is most likely to reach out to another party. Their end goal of negotiations is finding a willing and able third country that will resettle one or multiple detainees while adhering to a strict set of criteria. The criteria for resettlement have been codified in the NDAA for fiscal year 2011, which have been reinstated in the NDAAs for every following year:

“… [T]he government of the foreign country or the recognized leadership of the foreign entity to which the individual detained at Guantanamo is to be transferred

1. is not a designated state sponsor of terrorism or a designated foreign terrorist organization;
2. maintains effective control over each detention facility in which an individual is to be detained if the individual is to be housed in a detention facility;
3. is not, as of the date of the certification, facing a threat that is likely to substantially affect its ability to exercise control over the individual;
4. has agreed to take effective steps to ensure that the individual cannot take action to threaten the United States, its citizens, or its allies in the future;
5. has taken such steps as the Secretary determines are necessary to ensure that the individual cannot engage or reengage in any terrorist activity; and
6. has agreed to share any information with the United States that—
  (A) is related to the individual or any associates of the individual; and
  (B) could affect the security of the United States, its citizens, or its allies.”^23^

These strict requirements thus form the U.S. minimum position, of which they cannot significantly deviate. Therefore, if the U.S. were to give no other incentives their extreme of concessions (A2) lies very close to their minimum position (A1), leaving little room for a zone of agreement with a negotiation party. This has important implications for the negotiation process. As a result, the amount of viable potential resettlement country negotiation partners is limited, as some countries will likely never be able or willing to meet these criteria. This leaves the U.S. with two options for negotiation partners: (1) third countries that have a mutual interest in resettling the detainees (in those cases a zone of agreement already exists) and (2) third countries that may with the right incentives – be it through a carrot or a stick approach – be willing to make concessions to create a zone of agreement. Indeed, the latter is the very ‘incentive structure’ that Congressman Cellar in 1950 referred to when he warned for the hypothetical situation in which the U.S. could ‘persuade’ the French Government, under threat of having Marshall plan aid cut off, to resettle undeportable undesirables from the U.S.

A review of publicly available information about past third country resettlements suggests that U.S. negotiation partners appear to have had different motives to accept detainees. Conceptually, we suggest to differentiate countries that appear to be ideologically motivated – ‘the Idealists’ – from countries that are pragmatically motivated, ‘the Pragmatists’. It must be stressed that these categories are ‘archetypes,’ and that in in actual reality countries may be motivated by a combination of idealist and pragmatic motives. These categories are thus not necessarily mutually exclusive. We furthermore acknowledge that based on public information, it is highly complex to make proper assessments what motivates a government to take a certain course of action. Making this distinction is, however, conceptually useful in understanding the challenges and possibilities relevant to each third country resettlement negotiation.

### Idealists

**Fig. 8:**
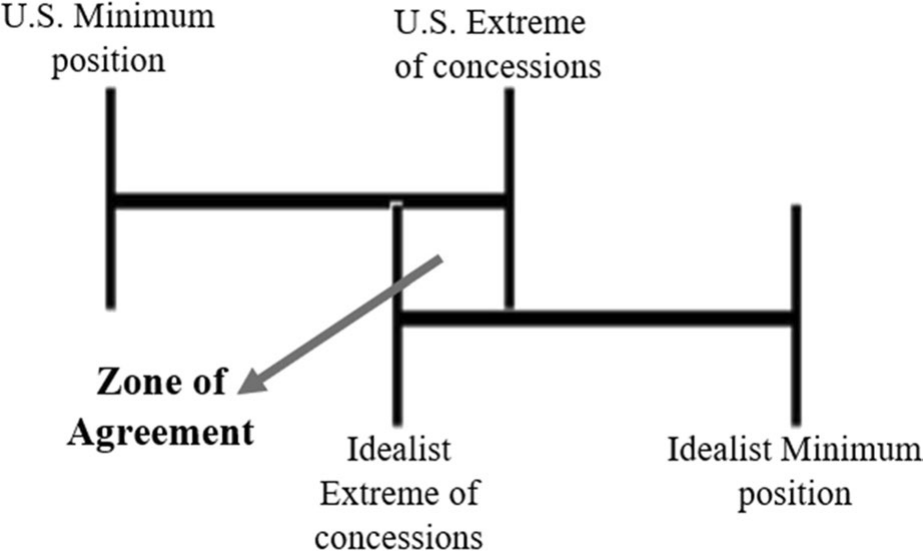
When the U.S. and Idealist’s interests are mutual and the Idealists country fulfils the U.S. criteria, there is a zone of agreement

**Fig. 9:**
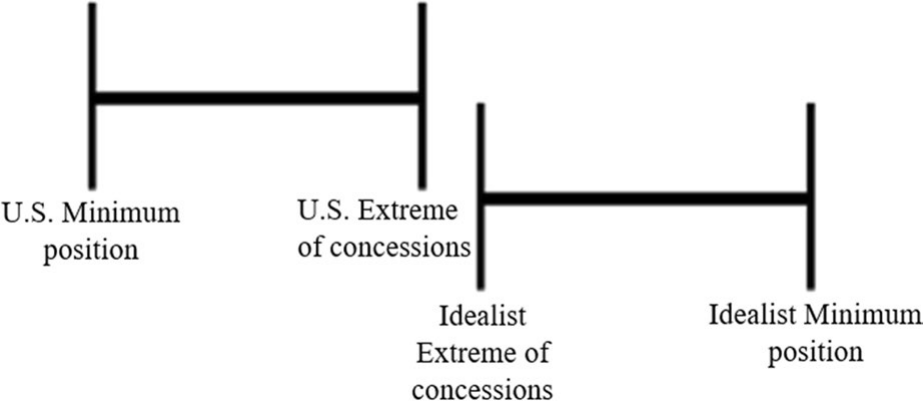
When the U.S. and Idealist’s interests are mutual, but the Idealist country cannot fulfil the U.S. criteria, there is no zone of agreement

Countries are considered to be ‘Idealists’ when they substantiate their willingness to resettle detainees by referring to ideological rationales. They, for example, publicly refer to a wish to protect human rights in current-day society as their motivation to take in a number of detainees, and function as advocates for resettlements in their respective region and beyond. Thus, the Idealists and the U.S. have a mutual interest in realizing third country resettlements. This creates a favorable situation for negotiation, in which starting positions are relatively close and few incentives may be necessary to come to an agreement. What can be a challenge to such negotiations is that the Idealist country still needs to meet the criteria for resettlements put forward in NDAA legislation. However willing a country is to take in detainees, an agreement is not possible without meeting these strict criteria. These two negotiation situations are visualized in Figs. 8 and 9.

Illustrations of countries which clearly expressed idealist motives to resettle detainees are Portugal and Uruguay. Portugal was one of the first countries to resettle cleared Guantánamo detainees and was an important advocate in compelling other European countries to follow suit. On the 10^th^ of December 2008, on the 60^th^ anniversary of the Universal Declaration of Human Rights, Portugal announced its willingness to resettle detainees in a letter to other EU leaders and urged them to join as well pointing out the consensus on the need to close Guantánamo:

“The time has come for the European Union to step forward. As a matter of principle and coherence, we should send a clear signal of our willingness to help the US government in that regard, namely through the resettlement of detainees. As far as the Portuguese government is concerned, we will be available to participate” [53].

It has been argued that for many members of the Portuguese political elite, the desire to close the camp is deeply personal. Portugal's former dictatorship, in power between 1932 and 1974, used to torture its political opponents and members of the current government as well as the opposition include victims of that torture. Former President Mario Soares, himself a victim of torture, referred to Guantanamo as “the scandal of all scandals.” Domingos Santos, longstanding leader of Portugal's Communist party and previously abused by the country’s secret police during the junta, was quoted saying that Portugal should accept former Guantanamo Bay detainees “on grounds of human rights because Guantanamo is a cancer which is afflicting society” [54].

Uruguay, on the other hand, agreed to take in six detainees in 2014, mainly on the initiative of its former president José Mujica, a former guerilla with the left-wing Tupamaro movement who also personally endured the hardships of being a political prisoner. While some of his critics have pointed out his motivations might not be purely idealistic,^24^ Mujica himself has always maintained that humanitarian concerns were the sole reasons for his willingness to accept the detainees:

‘After some arrangements we accepted, because today and always, with the exception of the painful years of the dictatorship, Uruguay has been a country of refuge and for us this is a question of principle. … We cannot ignore the formidable tragedy of people who spent 12, 13 years without any contact with the outside world and detained without a proven cause or seeing a prosecutor or judge. Without any kind of assurances. It is a disgrace for humankind [57].’

Leaked diplomatic cables have furthermore confirmed that Mujica appears to have made his offer purely out of personal principle, and an American official confirmed that there was no money involved [58].

While on paper resettlement agreements with the Idealists seem to be a solution to the problem of cleared but unreturnable detainees, in practice the assistance of Idealists – if such a category even exists in the realm of international relations – proved to only solve part of the problem. Only very few resettlement countries have supported resettling efforts as publicly and vocally as Portugal and Uruguay. Furthermore, even the few countries that presented themselves as Idealists have only been willing or able to take in a limited number of detainees. Therefore, the US has been in dire need to more actively ‘incentivize’ other possible negotiation partners to realize all necessary third country resettlements.

### Pragmatists

**Fig. 10:**
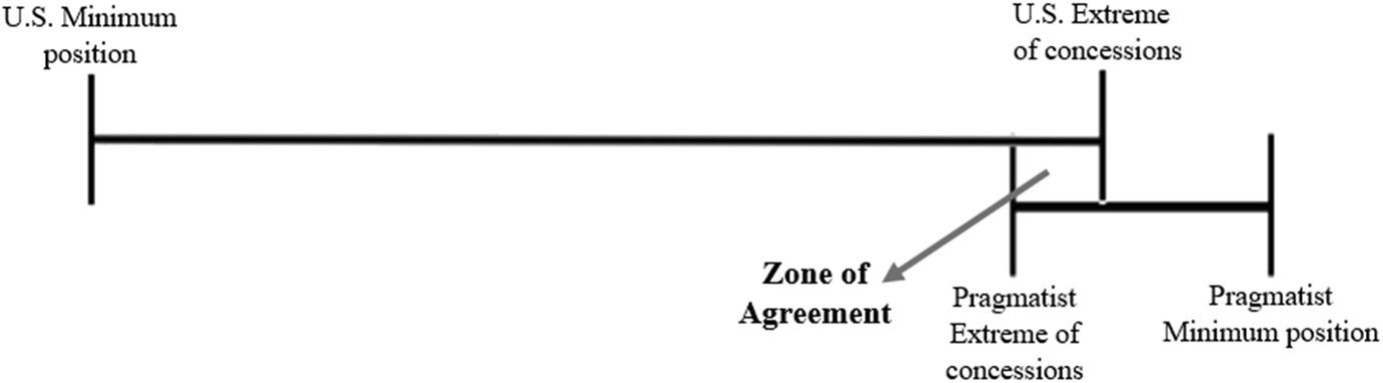
When the U.S. is willing and able to meet the Pragmatist’s demands asked in return for resettlements, there is a zone of agreement

**Fig. 11:**
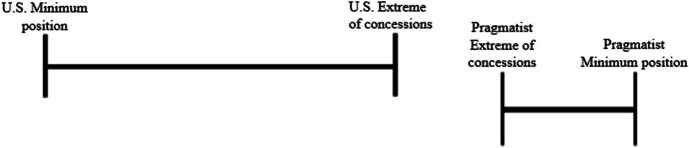
When the U.S. is unwilling or unable to meet the Pragmatist’s demands asked in return for resettlements, there is no zone of agreement

Countries are considered ‘Pragmatists’ when they do not express an intrinsic interest in resettling detainees, but seemingly have come to do so anyway after negotiating certain ‘incentives’. These incentives are needed as the Pragmatists and the U.S. do not have a mutual interest in resettling detainees, and therefore their minimum positions lie far apart. As mentioned earlier such incentives can be of a collaborative or competitive nature. Which incentives are effective in creating a zone of agreement is highly dependent on the specific Pragmatist country’s interests and their relationship with the U.S. Therefore, there is no one-size-fits-all negotiation strategy. The challenge for Pragmatist-U.S. negotiations is that the U.S. might not always be willing or able to meet the expectations in return for resettlement of the Pragmatists. Figs. 10 and 11 illustrate the possible outcomes of such negotiations.

While all negotiations with potential resettlement countries take place under strict confidentiality, leaked U.S. diplomatic cables up to 2010, as well as media reports, do give some insight in the types of incentives that have been on the table during such negotiations. Collaborative as well as competitive negotiation strategies seem to have been applied.

#### Financial incentives

Financial incentives seem to have been involved in practically all cases, first of all to compensate for relocation expenses. For example, the cables show that the Maldives were told by the Special Envoy that other resettlement states had received $25.000 to $85.000 per detainee to cover temporary living expenses and other costs, and should the Maldives accept detainees it could expect compensation towards the high end of that range.^25^ Bulgaria was presented with similar numbers during negotiation, namely $50.000 to $80.000 per detainee.^26^ Such monetary compensation sometimes is accompanied with other – much more relevant and substantial – monetary pledges focused on development aid and assistance. Kiribati, for example, was offered a total of $3 million if they were to take in the 17 Uyghur detainees that remained in Guantanamo in 2008, to be invested into assistance projects involving culvert and desalinization efforts.^27^ Similarly, Palau was also approached for resettling the same 17 Uyghur detainees and was reportedly offered up to $200 million in development, budget support and other assistance in return [59].^28^

#### Political favors and support

Apart from monetary incentives, more intangible concessions such as political favors and support also played a role in these negotiations. For example, during negotiations Bulgaria expressed the hope that in return for helping with the Guantanamo issue, the U.S. would assist in granting Bulgarian citizens Visa Waivers to the U.S. and deepen law enforcement relations in the future.^29^ The Maldives during the negotiations alternatively brought up the issue of an upcoming donor conference and U.S. support in gaining International Monetary Fund assistance. The leaked cables state that the Special Envoy replied that “*while there was of course no linkage between these issues and his own work*”, he would certainly pass on these points to Assistant Secretary Blake for South and Central Asian Affairs and would in addition “*make sure that President Obama knew not only of this decision on the detainees, but also more about the recent democratic developments in the Maldives, and the great story which had developed there*”.^30^ Other countries seem to have used resettlement negotiations to appease or strengthen their relationship with the U.S., like Switzerland, who offered to take in two detainees not long after the U.S. found out that major Swiss banks had aided rich Americans to evade taxes [62]. It has furthermore been argued that Albania’s aspirations to join NATO explained why it resettled Guantanamo Bay inmates already in 2006 [63].

Although the details of most resettlement talks and agreements remain confidential, the above illustrates the wide range of topics discussed and concessions involved in third country resettlement negotiations. Countries that have a lot to lose or gain can arguably be incentivized more easily. In this regard it is not surprising that a considerable number of the U.S.’ negotiation partners have been small states, who might consider resettlement as an attractive opportunity to gain political and monetary favors. This could also explain the strong presence of important strategic and military partners such as Oman, the UAE and Saudi Arabia among resettlement countries, as they arguably stand to lose a lot more by damaging relations with the U.S. than other countries. Overall, what makes a country (un)willing to negotiate depends on a variety of factors, as will be discussed in the next paragraph.

## Dynamics of the negotiation process

A multitude of factors determines the dynamics of each resettlement negotiation, influencing if and when third countries are willing to negotiate and what the minimum position and extreme of concessions of both the U.S. and the resettlement country are. As the nature of these factors can change over time, so can the nature of a negotiation process. While there are many potential factors of influence, the most important ones will be discussed below: the domestic politics and interests of the U.S., the domestic politics and interests of potential resettlement countries, and third-party interference.

### Domestic politics and interests of the U.S.

As the U.S. is usually the party that instigates negotiations, the dynamics of resettlement negotiations are first and foremost influenced by U.S. government’s stance. As discussed above the U.S. strategy for third country resettlement has not been one and the same over the years, and depends a lot on the domestic political situation. The number of resettlements over the years strongly correspond with the views of the sitting administration on the necessity of closing Guantanamo, with the vast bulk of resettlements being realized under the democratic Obama government.

Most of the resettlements under the Obama administration took place close to the beginning and the end of his term, with a significantly low number of resettlements taking place in 2011-2013. This gap was followed by a spike in resettlements, of which the vast majority was taken in by Oman, the UAE and Saudi Arabia, all of which can be qualified as authoritarian regimes. Cable leaks show that the U.S. already approached Oman and the UAE for resettlement assistance as early as 2009,.^31^ The cables suggest that at that point these countries were, given the then offered conditions, reluctant to take in detainees. As there is very little publicly available information regarding the resettlement negotiations after 2010, we cannot assess what had changed the situation by 2014-2016. Perhaps the U.S. by that time was willing to present more favorable incentives, perhaps it was willing to put these countries under more pressure, or – as discussed in the section below – perhaps the domestic political context changed.

### Domestic politics and interests of the potential resettlement country

Where the U.S. negotiation strategy is influenced by their domestic politics, the same goes for potential resettlement countries. Resettling detainees is generally considered as politically controversial. As the position of political leaders in democracies depends on public approval, they are usually only willing to make a controversial move such as taking in another country’s undesirable detainees when they have some leeway in taking such decisions without significantly dropping their approval ratings, and if they can sufficiently substantiate the benefits of that decision. Such domestic political interests have prevented successful resettlement negotiations in multiple cases. For example, in the fall of 2009 Lithuania backed out of its resettlement agreement following a domestic controversy around reports of a secret CIA jail in the country [64]. Leaked diplomatic cables also show that countries such as Slovenia and Panama both lacked the political will or courage to engage in resettlement negotiations as they had both been involved in controversial policy decisions regarding a border dispute and a pardon.^32^

Fears over the effects of the domestic response to a resettling agreement are not ungrounded. After the Premier of Bermuda Ewart Brown announced he had accepted four Uyghur detainees onto the island in secret, its parliament responded by considering a motion of no confidence [65]. Furthermore, dismayed citizens responded through a protest march against the Premier’s leadership [66]. Despite the controversy Brown eventually survived the vote of no confidence and fully served his term until October 2010 [67]. It is important to note that much of the controversy in this case stemmed from the fact that Brown agreed to take in the detainees without consulting his ministers, cabinet, its governor and UK authorities,^33^ and had been insincere towards parliament about the pre-transfer vetting process [65, 68]. Nonetheless, the case demonstrates that if the issue of Guantanamo detainee resettlement is politically not handled with utmost care, it can create political upheaval that potentially have significant repercussions.

Countries may also consider cooperating if they fear refusing to accept resettled detainees would damage their relations with the U.S. As U.S. support can be decisive in attaining goals out of national interest, this influences negotiation dynamics as well. For example, the damage to Dutch-U.S. relations caused by the standing refusal of the Netherlands to resettle detainees in 2015 sparked public debate as it came at a time where the Netherlands needed U.S. support. The country’s prosecution office was in an advanced stage of its MH17 plane crash investigation and expected U.S. support would be key in getting hold of the perpetrators in the near future. Furthermore, the Netherlands hoped to obtain a non-permanent seat in the UN Security Council elections in 2016, for which U.S. backing seemed essential as well [69]. While in the end the Netherlands did not take in any detainees despite this pressure, this case illustrates how domestic political pressure can impact resettlement negotiations.

Naturally, as issues of public approval are less relevant to countries that have an authoritarian leadership, such negotiations take on a different nature. A lack of pushback in the domestic political sphere leaves more opportunities to create a zone of agreement during negotiations, and furthermore allows these states to accept a higher number of detainees without leading to political controversy. Thus, the ability of authoritarian regimes to resettle Guantanamo detainees is significantly higher than that of democracies. As a result, negotiation dynamics are likely less dependent on factors outside of U.S. control, and more dependent on the – competitive or collaborative – incentives the U.S. is able and willing to use to create a zone of agreement. With the right incentives, the U.S. and an authoritarian government are therefore significantly more likely to come to an agreement. This might explain why, as presented earlier in Fig. 5, a disproportionately high number of detainees were resettled to countries with authoritarian regimes.

### Third party interference

Apart from domestic politics in the US and the potential resettlement country, at least two other sets of actors may also significantly influence the dynamics of resettlement negotiations: supranational, inter- and non-governmental organizations on the one hand, and countries of origin on the other. As to the first set of actors, relevant supranational and intergovernmental organizations such as the European Parliament,^34^ and the Organization for Security and Co-operation in Europe,^35^ have actively called upon member states to resettle Guantanamo Bay detainees. In addition, a large variety of human rights NGOs have also actively lobbied governments to consider taking in detainees, providing information and support in realizing successful third country resettlements and providing individual support to resettled detainees. Some of the most active NGOs in lobbying efforts include Human Rights Watch,^36^ Human Rights First,^37^ Reprieve,^38^ and CAGE.^39^ Some of these efforts have even been supported by former Guantánamo detainees themselves, such as Moazzam Begg, who after his return to his home country of the U.K. became a public commentator on Guantánamo and has been strongly involved with lobbying activities of Reprieve and CAGE [71]. While it is challenging to determine the exact influence lobbying has had on the dynamics of resettlement negotiations, it is reasonable to assume that the information and support lobbying organizations provided has affected the decision making process of potential resettlement countries’ and thereby their stance during negotiations.

Finally, countries of origin of the resettled detainee – or powerful actors in these countries – may influence resettlement negotiations. The decision to take in (certain) detainees can also negatively affect relations of the resettlement country with the detainee’s country (or countries) of origin. As a result, discouragement and pressure from countries of origin can influence a potential resettlement country’s willingness to take in detainees. Especially when relations with that country are highly valued, this can heavily affect negotiations. An example of the influence of a such a powerful actor is the previously discussed role of the Taliban in the resettlement negotiations of five Afghan detainees to Qatar. Another example can be found in the leaked diplomatic cables, through which Portugal’s unwillingness to consider Tunisian detainees – after pressure from the Tunisian government – was relayed to the U.S.^40^

However, the most notorious example of the influence a country of origin can have on negotiation processes are China’s efforts to prevent potential resettlement countries of Uyghur detainees from taking them in. As discussed previously, all Chinese Uyghur detainees were suspected of being part of the ETIM, a group that China considers to be a terrorist organization. Therefore, China has always urged the Uyghur detainees are terrorist suspects that should be returned to face justice domestically. The U.S. instead opted for resettling the Uyghur detainees out of human rights concerns, but finding them a new home proved to be a challenge due to Chinese pressures on potential resettlement countries. Leaked cables demonstrate many countries declined U.S. requests for resettlement assistance for Uyghur detainees, with countries such as Germany, Finland, Portugal, the Czech Republic and Sri Lanka specifically stating they could not accept Uyghurs because of the expected strain this would put on their bilateral relations with China.^41^ Although in the end the U.S. was able to resettle all Uyghur detainees, the resettlement countries have been under a lot of scrutiny by China. Albania was the first to resettle Uyghur detainees, welcoming five of them in May 2006 after the U.S. reportedly spent 18 months finding a country willing to accept them. This became a rough patch in Albanian-Chinese relations, and while Albania did not respond to China’s requests to extradite the Uyghurs, leaked cables show Albania was unwilling to resettle more Uyghur detainees afterwards [63].^42^ Likewise, China has been critical of all Uyghur resettlements, often denouncing them publicly after their transfer.^43^

## Third country resettlements, a success story?

While third country resettlements have enabled the release of 150 detainees from the Guantánamo Bay facility, the question remains whether resettlement can be considered a successful approach to deal with the issue of undesirable and unreturnable non-citizens. The answer to this question depends on which perspective is taken and strongly differs per involved actor, as we will discuss below.

### The U.S.

The U.S. – in particular Obama’s administration – would arguably consider the resettlement process successful if it clears detainees out of the Guantánamo facility, without the resettled individuals posing a continued threat to U.S interests, and without them being subjected to torture.

In this respect it is relevant to note that there are no indications that any of the resettled individuals has been tortured or otherwise persecuted. As to posing a threat to U.S. interests, U.S. government has been monitoring if returned or resettled detainees pose a security threat. The U.S. Director of National Intelligence reported that as of 15 July 2019, 124 of all 729 transferred Guantánamo detainees (including those returned to their country of origin) had been confirmed to have reengaged in terrorism, with a further 102 detainees suspected of reengagement. Only 9 of the confirmed and 20 of the suspected reengaged detainees were transferred after January 2009, when Obama took office.^44^ As 94% of all third country resettlements took place under the Obama administration, the number of resettled detainees reengaging in terrorism is likely to be relatively low, although not insignificant. However, as mentioned before, the office responsible for overseeing detainees post-transfer was closed down by the Trump administration in 2017. As a result, there has been a lack of monitoring of recidivism in the recent years; hence the data on reengaged detainees after third country resettlement might be incomplete. While reengagement after resettlement would undermine the success of the policy, the lack of monitoring makes it impossible to draw any definite conclusions on the successfulness of third country resettlements from the U.S. perspective.

### Resettlement countries: Idealists

From the Idealist perspective, third country resettlement is arguably a relative success by the mere accomplishment of having detainees released from the Guantanamo Bay facility. As the case of Uruguay demonstrates, evaluations may however change over time, for example if the reintegration process of resettled detainees is problematic.

While president Mujica was a strong proponent of third country resettlement when six detainees were transferred to Uruguay, just a year later he became very critical of the resettled detainees. Throughout the year they had publicly expressed dissatisfaction about the, in their eyes, limited support offered by the Uruguayan government. For weeks five ex-detainees even went on hunger strike and camped outside the U.S. Embassy in Montevideo demanding more financial assistance [74, 75]. Two detainees had stirred controversy by marrying Uruguayan women according to Islamic ritual without having a civil marriage first, as required by Uruguayan law. These controversies became even more pressing when one of them was by his wife accused of domestic violence, and the other was issued a restraining order after verbal threats to his wife [75, 76]. Following these incidents Mujica publicly expressed his disdain of the detainees’ conduct. April 2016 he stated: “*You cannot come to someone else's house with a different culture and expect to impose your beliefs, as Don Quixote said ‘wherever you go, do as you see.’ It is elementary*’ [75].^45^

Furthermore, Mujica added that their conduct was not only problematic for Uruguay, but for the larger effort to find a new home for all cleared but unreturnable Guantánamo detainees:

“The behavior of the detainees that were taken in is lousy. It shows an absolute lack of solidarity with those who that are still there [in Guantánamo]. Because, if they had presented a different image they would have made it easier for others to be released. The only thing they did was making three or four governments in Latin America who were about to agree to similar measures retreat. Who did they harm? The other prisoners at Guantánamo” [77].^46^

The whole episode caused Mujica to even contemplate on the much more banal, pragmatic and non-idealist motivations his government apparently had (also) had for taking in the detainees: “*In order to sell a few kilos of oranges to the United States I had to take in five crazy people from Guantánamo”* [74].^47^

Uruguay is one of the few resettlement countries, let alone those who expressed such ideological motives after accepting the detainees, where (ex)government representatives have been so open and vocal about their experiences with resettlement. Assuming Uruguay’s experiences are not representative of all other (partially) idealistically motivated governments, it seems that Idealists would generally consider the third country resettlements somewhat effective in preventing further human rights abuses in Guantánamo. Whether they regard resettlements as a success is likely to differ from case to case.

### Resettlement countries: Pragmatists

For the Pragmatist a resettlement process would arguably be judged positively if the benefits gained or losses avoided by taking in detainees outweigh any negative repercussions of this decision. As most resettlement countries have not publicly commented on their experiences, it is difficult to make any definite conclusions regarding the successfulness of third country resettlements from their perspective. When resettlements are publicly discussed, reporting is usually focused on negative experiences, such as the previously discussed cases of Chinese dismay influencing international relations after resettlements in Albania and Palau among others, and the Bermudan president risking to lose his position over resettlements to the island nation. In addition, countries that agreed to resettle detainees only temporarily, have reported problems when the resettlement period came to an end. Both Palau and Ghana, for example, reported challenges in finding new resettlement countries for the detainees they agreed to resettle temporarily [61, 78]. It is unclear if, and to what extent, the U.S. offers support in these instances. While Palau was eventually able to ‘re-resettle’ all detainees they temporarily hosted [79], the Ghanaian government – after hefty political debates and a 2017 Supreme Court decision which declared the resettlement deal unconstitutional – ultimately allowed its detainees to remain in Ghana under refugee status [80, 81].

The above examples point towards a negative evaluation of the successfulness of third country resettlements from the perspective of a number of Pragmatists. However, it should be noted that more successful resettlement experiences are likely to garner less media attention, and therefore negative experiences might overshadow the positive in public debate. With regards to non-democratic regimes it may actually be very difficult to find any public information about negative experiences. Ultimately, the perceived successfulness will likely be strongly influenced by how each individual resettlement plays out on the long term in a resettlement country. Similar to the Idealist countries, whether Pragmatist countries regard resettlements as a success is therefore likely to differ from case to case.

### Resettled detainees

Finally, the resettled detainees themselves have their own perspective on the successfulness of third country resettlements. While the fact that they are released from the Guantánamo facility would in itself seemingly be a success, their assessment of the resettlement will also depend on what their life after resettlement looks like. As each of the 150 detainees have a unique resettlement story, it goes beyond the scope of this article to extensively discuss their experiences. Nonetheless, some preliminary research reveals the wide variety in experiences of resettled detainees.

First of all, there are large differences in the time detainees spent waiting in Guantánamo for a country to take them after clearance, ranging from a few months up to several years. Second, what life after resettlement looks like depends on the resettlement country. Each government has different policies on the legal status, rights, freedoms and support provided to resettled detainees. Some resettlement countries like Ireland, Bermuda and Latvia are reported to have offered a legal status, options for family reunification, financial assistance and other support [82–84]. Other resettlement countries, such as Albania, reportedly offer very limited support to resettled individuals [85]. Socio-cultural differences between resettled detainees and their resettlement countries can also impact the resettled detainee’s life after resettlement. Resettled detainees have little say in which country they are resettled to, and some have been reported to struggle with adapting to the local life. For instance, a Syrian detainee resettled to Cape Verde was fully reliant on an interpreter to carry out daily activities [86] and an Egyptian detainee resettled to Slovakia reportedly experienced difficulties adjusting to the country where he has “*no family, no wife, no children and no Muslims*” [87].

In the end, it seems that the experiences of resettled Guantanámo detainees are very diverse. Some detainees have been relatively content with their resettlement, such as Algerian ex-detainee Lakhdar Boumediene:

“Today, I live in the Provence with my wife and children. France has given us a home, and a new start. I have experienced the pleasure of reacquainting myself with my daughters and, in August 2010, the joy of welcoming a new son, Yousef. I am learning to drive, attending vocational training and rebuilding my life” [88].

Such positive stories are rather exceptional though. Many have expressed dismay with their situation, such as Lotfi Bin Ali, a Tunisian ex-detainee resettled to Kazakhstan. He lives in an apartment, can cook his own meals, but family reunification is not possible and his whereabouts are very closely monitored:

“This is life in Kazakhstan. Second Guantanamo. … [In Guantanamo] we got our medication, food, and everything. Here, I have to come to the center [of the Kazakhstan Red Crescent Society] and ask them to bring me medication. … How long can I live like this? Is this life better than when we were handcuffed?” [89].

Finally, there are those whose voices cannot be heard. Most of the 23 mainly Yemeni detainees which Obama’s administration in 2015 and 2016 managed to resettle to the UAE jumped out of the frying pan into the fire. They are reportedly still held in its infamous Al-Razeen prison, locally known as ‘the Guantanamo of the UAE’ [90, 91].^48^

## Conclusion

States are increasingly confronted with undesirable non-citizens who are considered to pose a threat to national security, but cannot be deported to their country of origin (UBUs). In light of the wider discussion on the merger between national security and migration law and the question how states can deal, should deal or do deal with UBUs, this article described the resettlement process of unreturnable Guantanamo Bay detainees. After presenting an overview which states resettled cleared and unreturnable Guantanamo Bay detainees, it analyzed why some states resettled these UBUs while others have not and discussed to what extent – from the perspective of the most relevant actors involved – the resettlement process can be considered a success or not.

When Obama in 2009 pledged to close Guantanamo Bay, political representatives of liberal democracies were typically the first to support these plans. This article shows that when push came to shove, few democratic governments were actually willing to help out the U.S. administration by resettling cleared but unreturnable Guantanamo Bay detainees. Only 24% of the countries that resettled detainees can be considered ‘full democracies,’ while these countries only resettled 13% of the in total 150 resettled detainees. Long time Anglo-Saxon allies like Canada, Australia and even the United Kingdom – with its ‘special relationship’ with the U.S. and being the first European country to join the ‘coalition of the willing’ in supporting the 2003 Iraq invasion – did not resettle a single unreturnable Guantanamo Bay detainee. It is understandable that governments were not very keen on helping out the U.S. Administration. As U.S. Congress fully prohibited any transfers or releases of detainees to U.S. soil, it is hard to make a case why another country should accept them. For democracies, merely informing parliament about the initiation of resettlement negotiations could already cause domestic political consternation. Internationally, resettling detainees could have negative political or economic repercussions, in particular when it concerned resettlement of Chinese Uyghurs. A few – seemingly – idealistic leaders aside, this may explain why few democracies were willing to resettle significant numbers of detainees. Another explanation why democratic countries resettled relatively few detainees, may have been the strict criteria U.S. government set. As discussed above, one of these conditions was that the government of the resettlement country “maintains effective control over each detention facility in which an individual is to be detained if the individual is to be housed in a detention facility.” This suggests that the U.S. with regards to some detainees – like those who are considered to pose a continuing security threat to the U.S. – requires they are detained after resettlement, without having been convicted of a crime. For understandable reasons, liberal democracies could not accept this condition.

Apart from making moral appeals to full democracies the U.S. shares political values with, it therefore also tried to find ‘zones of agreement’ by incentivizing more pragmatically oriented governments through negotiations, thereby using a carrot-or-stick approach. Eventually, this strategy proved the most successful. The Obama administration mainly relied on relatively ‘weak’ nations such as Bermuda or Palau for resettling Uyghur detainees who could not be deported for human rights concerns. Highly authoritarian regimes such as the UAE, Oman and Saudi Arabia resettled a considerable number of detainees of other nationalities who could not be deported for human rights concerns or were considered to pose a continuing security threat. With the help of these countries many cell blocks in Guantanamo Bay were emptied before the Trump government took over. The exact content of the ‘quid pro quo’ is unknown due to confidentiality of the resettlement agreements, but there are indications that assisting the U.S. in resettling detainees led to short term relatively tangible financial, military or political gains or, on the longer run, rather intangible political ‘goodwill’.

This has resulted in a somewhat cynical situation. In closing the ‘cancer which is afflicting society’ – to borrow the words of earlier quoted Portuguese Communist party leader Domingos Santos – Nobel Prize winning President Barack Obama had to rely heavily on the cooperation of authoritarian states considered to be human rights violators themselves. Some cleared and ‘resettled’ detainees are still detained without due process; not in a U.S.-ran military base on Cuba, but in a prison far off in the UAE desert instead. Indeed, the realpolitikal powerplay to ‘outsource’ U.S. governments’ UBU-problem that Congressman Cellar already warned for in 1950, has 70 years later become a reality.

Whether the different actors involved consider the resettlement process a success is difficult to establish. A lack of monitoring makes it impossible to draw any definite conclusions as to whether the resettled individuals pose a security threat to the U.S. The experiences of the resettlement countries vary significantly. Some governments are outrightly negative about the process, while others are mainly silent. It is to be expected that more ‘successful’ resettlement experiences are likely to garner less media attention. The experiences of the resettled detainees are very diverse as well, with some expressing to be relatively content, while others are complaining heavily about the many restrictions they continue to experience. More research is needed to make a proper assessment in this regard.

There are some important lessons to be learned from this case study. While today a relatively small number of men continues to be detained at Guantánamo, new situations in which foreign alleged terrorists are held in detention facilities without due process have emerged. Eerily similar to Guantanamo Bay, is the case of foreign fighters detained in Kurdistan. After the defeat of IS in the northern regions of Syria, alleged IS-fighters have been detained in ‘pop-up prisons’ in the region. Reportedly, more than 10,000 men of over 50 nationalities (mostly Syrian and Iraqi, but also including individuals from Europe, Africa, Asia and North America) are detained in such makeshift prisons, while their wives and children are held in separate camps [93]. A significant number of these detainees has claimed to have no connection to IS, and to be held unjustly. The conditions in the overcrowded prisons deteriorate each day, and have become even more worrying with the rise of the coronavirus pandemic [93]. However, Kurdistan does not have an appropriate legal system in place to deal with the prisoners, and the establishment of an international tribunal that would potentially take on the enormous task of trying these detainees seems at the least years away [94]. Therefore, most (foreign) fighters will likely be stuck in these prisons for a yet undetermined period of time.

Assuming some of these detainees will someday be cleared for release, what can we expect to happen to them? This article learns us not to expect much of western democracies helping out. In fact, some democracies like Great Britain, the Netherlands, Belgium and Denmark were the first to revoke the nationality of their foreign fighters that held dual citizenship, thereby complicating any future deportations [5, 95]. If the Kurdish authorities, possibly with the help and leverage of the U.S., ever try to resettle any of these detainees, their best bet may be to press or lure some small island nations or authoritarian regimes into cooperation. In particular the resettlement to countries with highly authoritarian regimes can, from a human rights perspective, be problematic. The transfer of the more than 20 Guantanamo detainees to a UAE prison in this respect sets a concerning precedent. This type of resettlement certainly is not the ‘sensible or sustainable solution’ to the UBU-problem [6] that is so direly needed.

## Appendix: Detainees listed as resettled in Guantánamo Docket but excluded from the database

**Table:**

Name	ISN	Nationality registered in the Guantanamo Docket Database	Country of Resettlement registered in the Guantanamo Docket Database	Reason for exclusion	Sources
Sameur Abdenour	659	Algeria	Britain	Was a legal resident of the U.K. under refugee status before detention, and therefore released to the U.K.	• https://www.bbc.com/news/uk-39115761 • https://www.theguardian.com/uk/2007/dec/08/guantanamo.usa
Hadj Boudella	10006	Algeria & Bosnia and Herzegovina (dual)	Bosnia and Herzegovina	Possessed dual citizenship to Algeria and Bosnia and Herzegovina. While Bosnia stripped him of his Bosnian citizenship in 2001, this decision was later reversed. Was released to Bosnia, a country he held citizenship to at the time of release, where he was reunited with his family and friends.	• https://css.ethz.ch/en/services/digital-library/articles/article.html/94887 • https://www.reuters.com/article/us-guantanamo-bosnia/u-s-sends-three-guantanamo-men-home-to-bosnia-idUSTRE4BF5BG20081216 • https://www.rferl.org/a/Bosnian_Detainees_Return_Home_After_Seven_Years_In_Guantanamo/1360902.html
Mustafa Ait Idr	10004	Algeria & Bosnia and Herzegovina (dual)	Bosnia and Herzegovina	Possessed dual citizenship to Algeria and Bosnia and Herzegovina. While Bosnia stripped him of his Bosnian citizenship in 2001, this decision was later reversed. Was released to Bosnia, a country he held citizenship to at the time of release, where he was reunited with his family and friends.	• https://css.ethz.ch/en/services/digital-library/articles/article.html/94887 • https://www.reuters.com/article/us-guantanamo-bosnia/u-s-sends-three-guantanamo-men-home-to-bosnia-idUSTRE4BF5BG20081216 • https://www.rferl.org/a/Bosnian_Detainees_Return_Home_After_Seven_Years_In_Guantanamo/1360902.html
Mohammed Nechle	10003	Algeria & Bosnia and Herzegovina (dual)	Bosnia and Herzegovina	Possessed dual citizenship to Algeria and Bosnia and Herzegovina. While Bosnia stripped him of his Bosnian citizenship in 2001, this decision was later reversed. Was released to Bosnia, a country he held citizenship to at the time of release, where he was reunited with his family and friends.	• https://css.ethz.ch/en/services/digital-library/articles/article.html/94887 • https://www.reuters.com/article/us-guantanamo-bosnia/u-s-sends-three-guantanamo-men-home-to-bosnia-idUSTRE4BF5BG20081216 • https://www.rferl.org/a/Bosnian_Detainees_Return_Home_After_Seven_Years_In_Guantanamo/1360902.html
Juma Mohammed Abdul Latif al Dosari	261	Bahrain	Saudi Arabia	While listed in the Guantánamo Docket as being solely Bahraini, he holds dual citizenship to Bahrain and Saudi Arabia. He was therefore released to Saudi Arabia, where he reunited with his family.	• https://www.cage.ngo/interview-juma-al-dossary • http://www.bahrainrights.org/en/node/1391 • https://www.latimes.com/archives/la-xpm-2007-dec-21-fg-rehab21-story.html
Abdurahman Khadr	990	Canada	Bosnia and Herzegovina	While accounts differ, it seems he was recruited by the CIA before his detention as an informant in the Guantánamo facility. After some months in the facility the CIA sent him to Bosnia for a new spy operation among mosques in Sarajevo. However, not long after he quit the CIA and moved back to Canada.	• https://www.pbs.org/wgbh/pages/frontline/shows/khadr/interviews/khadr.html • https://www.nytimes.com/2004/06/21/world/the-reach-of-war-guantanamo-memories-from-outside-the-wire.html • https://www.cbc.ca/news2/background/khadr/alqaedafamily6.html
Tariq Mahmoud Ahmed al Sawah	535	Egypt & Bosnia and Herzegovina (dual)	Bosnia and Herzegovina	Orignally obtained Bosnian citizenship after participating in the Bosnian war in the early 1990s, after which he stayed, married a Bosnian women and had a daughter with her. While he was stripped of his Bosnian citizenship in 2007 before his release to that country in early 2016, his case was still excluded from the database as its nature differs significantly from other third country resettlements.	• https://www.middleeasteye.net/features/life-after-guantanamo-former-egyptian-prisoner-stuck-bosnia-limbo • http://ba.n1info.com/Vijesti/a79754/Tariq-Al-Sawah.html • https://www.eurasiareview.com/04052017-life-after-guantanamo-egyptian-in-bosnia-stranded-in-legal-limbo-oped/
Binyam Mohamed	1458	Ethiopia	Britain	Was a legal resident of the U.K. as an asylum seeker before detention, and therefore released to the U.K., where he was reunited with his family. Afterwards he received permanent residency to Britain.	• https://www.bbc.com/news/uk-39115761 • https://www.theguardian.com/uk/2009/feb/23/binyam-mohamed-guantanamo-plane-lands • https://www.dailymail.co.uk/news/article-1319220/Binyam-Mohamed-stay-Britain%2D%2Dwants-kept-secret-Former-detainee-argues-story-amounts-torture.html
Bakhtiar Bamari	623	Iran	Afghanistan	While sources are scarce, it seems he was transferred to Bagram prison in Afghanistan, where he spent 6 months before being repatriated to Iran.	• http://en.ce.cn/World/Middleeast/200409/16/t20040916_1777429.shtml • https://www.kuna.net.kw/ArticlePrintPage.aspx?id=1489773&language=en • http://www.payvand.com/news/04/sep/1136.html
Bisher Amin Khalil al Rawi	906	Iraq	Britain	Was a legal resident of the U.K. with refugee status before detention, and therefore released to the U.K., where he was reunited with his family.	• https://www.bbc.com/news/uk-39115761 • https://www.nzherald.co.nz/world/news/article.cfm?c_id=2&objectid=10432211
Jamil El Banna	905	Jordan	Britain	Was a legal resident of the U.K. with refugee status before detention, and therefore released to the U.K. While upon release he was immediately arrested for an outstanding Spanish extradition warrant for suspected connections with a Spanish Al-Qaeda cell, he was released soon after as he was ruled unfit to stand trial in Spain. Herafter, he was reunited with his family in the U.K.	• https://www.bbc.com/news/uk-39115761 • https://www.theguardian.com/world/2008/mar/06/spain.uksecurity
Zaid Muhamamd Sa'ad al Husayn	50	Jordan	Saudi Arabia	Is listed as Jordanian the Guantanamo Docket. However, always claimed that he was technically born in Jordan but has Saudi parents, has a Saudi passport and lived there his whole life. However, both Saudi Arabia and Jordan did not identify him as a citizen of their country early on during his detainment. Nonetheless, it is registered he was released to Saudi Arabia in 2007, seemingly as a citizen of that country.	• https://www.nytimes.com/interactive/projects/guantanamo/detainees/50-zaid-muhamamd-sa-ad-al-husayn/documents/11
Omar Amer Deghayes	727	Libya	Britain	Was a legal resident of the U.K. before detention, and therefore released to the U.K. While upon release he was immediately arrested for an outstanding Spanish extradition warrant for suspected connections with a Spanish Al-Qaeda cell, he was released soon after as he was ruled unfit to stand trial in Spain. Herafter, he was reunited with his family in the U.K.	• https://www.bbc.com/news/uk-39115761 • https://www.theguardian.com/world/2010/jan/21/i-fought-to-survive-guantanamo • https://www.theguardian.com/world/2008/mar/06/spain.uksecurity
Ali Muhammad Abdul Aziz al Fakhri	212	Libya	Morocco	Was in secret CIA rendition from 2001 until he was sent back to Libya in 2006. He is suspected to have been transferred through at least Afghanistan, Egypt, Mauritania, Poland, Morocco, Jordan and then back to Afghanistan in late 2003. Records show he spend a few months at Guantánamo between September 2003 and April 2004 and was flown to Morocco afterwards for continued detention, explaining the registered transfer in the Guantanamo Docket. After this transfer there is a gap in knowledge, until he is thought to have been transferred from Afghanistan to Libya in 2006. He has seemingly never been outside of U.S. custody until this transfer back to his country of origin. He died in Libyan custody in 2009.	• https://www.therenditionproject.org.uk/prisoners/ibn-sheikh-al-libi.html • https://www.cage.ngo/ibn-al-shaykh-al-libi
Laacin Ikassrin	72	Morocco	Spain	Transferred to Spain with the goal of criminal prosecution, not resettlement. Eventually he was acquitted, but as Morocco revoked his nationality he remains in Spain without documentation. In 2016 he was indicted to 11,5 years in prison for Jihadism charges in Spain.	• https://elpais.com/diario/2005/07/19/espana/1121724026_850215.html • https://www.elmundo.es/elmundo/2008/10/09/espana/1223520814.html • https://www.elconfidencial.com/espana/2016-09-28/11-anos-y-medio-de-carcel-a-un-expreso-de-guantanamo-por-mandar-voluntarios-a-siria_1267271/ • https://www.lavanguardia.com/vida/20170505/422307994217/el-supremo-confirma-la-condena-de-11-anos-y-medio-de-prision-al-expreso-de-guantanamo-y-lider-de-la-brigada-al-andalus.html
Noor Aslaam	822	Pakistan	Afghanistan	While there seem to be no publicly availabe sources on the actual transfer, the Joint Task Force Guantanamo Assessment form shows he was approved for ‘transfer to the control of another government for continued detention.’ It is therefore likely that this is what happened when he was transferred to Afghanistan.	• https://www.nytimes.com/interactive/projects/guantanamo/detainees/822-noor-aslaam
Shaker Aamer	239	Saudi Arabia	Britain	Was a legal resident of the U.K. before detention, and therefore released to the U.K. where he was reunited with his family.	• https://www.bbc.com/news/uk-39115761 • https://www.theguardian.com/world/2015/oct/30/shaker-aamer-to-seek-damages-over-guantanamo-bay-incarcaration
Yaser Esam Hamdi	9	United States & Saudi Arabia (dual)	United States	First, transferred from Guantánamo to a U.S. prison, eventually released to Saudi Arabia on the condition he revoked his U.S. nationality and adheres to travel prohibitions. As he possesses both U.S and Saudi nationality, none of these instances concern third country resettlement.	• https://edition.cnn.com/2004/WORLD/meast/10/14/hamdi/ • https://www.nytimes.com/2004/09/22/politics/us-agrees-to-release-american-caught-with-taliban.html?searchResultPosition=4
Ahmed Khalfan Ghailani	10012	Tanzania	United States	Transferred to U.S. with the goal of criminal prosecution, not resettlement. Has been sentenced to life in prison.	• https://www.csmonitor.com/USA/Justice/2011/0125/Ahmed-Ghailani-gets-life-sentence-for-Al-Qaeda-bombing-of-US-embassies • https://www.bbc.com/news/world-africa-11485162
Adel Ben Mabrouk	143	Tunisia	Italy	Transferred to Italy with the goal of criminal prosecution, not resettlement. Was deported back to Tunisia after being sentenced time served.	• https://www.nytimes.com/2009/12/01/world/europe/01briefs-Italybrf.html • https://www.washingtonpost.com/wp-dyn/content/article/2009/11/30/AR2009113002950.html • https://www.jurist.org/news/2011/04/italy-deports-former-guantanamo-detainee-to-home-country-of-tunisia/
Mohamed Ben Riadh Nasri	510	Tunisia	Italy	Transferred to Italy with the goal of criminal prosecution, not resettlement.	• https://www.nytimes.com/2009/12/01/world/europe/01briefs-Italybrf.html • https://www.washingtonpost.com/wp-dyn/content/article/2009/11/30/AR2009113002950.html
Murat Kurnaz	61	Turkey	Germany	Born in Germany, was a legal resident before detention. He was released to Germany where he was reunited with his family.	• Five years of my life: An innocent man in Guantánamo – Murat Kurnaz

## Footnotes

1 For example, see Associated Press [9] & Margulies [10].

2 Different government institutions have been responsible for reviewing the detention status of Guantánamo detainees. In chronological order these were the Combat Status Review Tribunals, the Administrative Review Board, the Guantanamo Detainee Task Force and the Periodic Review Board. While the legal basis and mandates of these institutions differ, their general purpose has been to determine whether a Guantánamo detainee was to remain in open-ended detention or put on a list of detainees recommended for transfer/release [11–13].

3 Detainees cleared for release are those ‘judged a sufficiently low threat that they were sent to countries with no expectation of follow up’ ([15], p. 2). Detainees cleared for transfer are those assessed to pose a relatively higher threat, and are therefore ‘conveyed with the expectation that some process would be applied in the receiving nation to mitigate the threat they potentially posed’ (ibid., p. 2).

4 Such as Access to Information and Freedom of Information Requests, see: Walby and Larsen [17] and e.g. Singer [18].

5 As explained in the section ‘Patterns and trends in third country resettlements’ third countries do not only permanently, but also temporarily resettle ex-detainees. Temporary resettlement – according to our criteria –encompasses resettlement for a ‘substantial period of time’, meaning for a considerable number of months or years.

6 UN Convention against Torture and Other Cruel, Inhumane, or Degrading Treatment or Punishment, United Nations, 10 December 1984, 1465 U.N.T.S. 85.

7 In recent years the Uyghurs have received more international attention following reports of human rights abuses against the minority during detentions in so-called ‘re-education camps’ established by the Chinese government. For more information on this issue see e.g. Raza [22] & Finnegan [23].

8 More information about the contested origins of ETIM, see e.g. Lee [26] and Mumford [27]

9 5 of the Chinese Uyghur detainees were cleared and resettled in May 2006, thereby being the first Guantanamo detainees to ever undergo third country resettlement. The remaining 17 Uyghurs were all cleared at once on September 30 2008 by a Notice of Status to the U.S. District Court for the District of Columbia, see https://amlawdaily.typepad.com/amlawdaily/files/Notice.pdf (accessed 2 March 2020).

10 National Defense Authorization Act for Fiscal Year 2016, Pub. L. No. 114-92, 129 Stat. 726 (2015).

11 National Defense Authorization Act for Fiscal Year 2017, Pub. L. No. 114-328, 130 Stat. 2000 (2016).

12 Executive Order No. 13,492, 3 C.F.R. 203 (2009).

13 National Defense Authorization Act for Fiscal Year 2010, Pub. L. No. 111-84, 123 Stat. 2190 (2009).

14 National Defense Authorization Act for Fiscal Year 2011, Pub. L. No. 111-383, 124 Stat. 4137 (2011).

15 The countries that have taken in detainees are Albania, Belgium, Bermuda, Bulgaria, Cape Verde, El Salvador, Estonia, France, Georgia, Germany, Ghana, Hungary, Ireland, Italy, Kazakhstan, Latvia, Montenegro, Oman, Palau, Portugal, Qatar, Saudi Arabia, Senegal, Serbia, Slovakia, Spain, Switzerland, the United Arab Emirates and Uruguay. Although details of the resettlement schemes are not publicly available, a limited number of countries explicitly only offered temporary resettlement, allowing the detainees to stay until another country willing to resettle them permanently was found or until changes in the situation in the country of origin would allow for return. Palau, for example, in 2009 expressed to ‘temporarily’ host Chinese Uyghurs, without giving any specificities [38]. Just before the arrival of two Yemeni detainees to Ghana in 2009, Ghana’s Ministry of Foreign Affairs stated to have indicated “a readiness to accept them for a period of two years, after which they may leave the country” [39].

16 For example, 22% of all U.S. weapon exports between 2014-2018 were destined for Saudi Arabia, with a further 6,7% destined for the UAE, making them the 1^st^ and 3^rd^ largest importers of U.S. weapons [44]. Furthermore, Oman has been an important strategic partner of the U.S. in the region since 1980 (for more detailed info, see [45]), and U.S.-UAE relations were described as ‘the strongest relationship that the United States has in the Arab world today’ in 2014 by CENTCOM commander General Anthony Zinni [46]. Also, all three countries receive security cooperation support of the U.S. and host U.S. military bases and facilities within their borders [47, 48].

17 The Economist Democracy Index 2019 is available at https://www.eiu.com/topic/democracy-index

18 France, Germany, Ireland, Portugal, Spain, Switzerland and Uruguay.

19 Belgium, Bulgaria, El Salvador, Estonia, Ghana, Hungary, Italy, Latvia, Serbia and Slovakia.

20 Albania, Georgia, Montenegro and Senegal.

21 Kazakhstan, Oman, Qatar, Saudi Arabia and the UAE. Due to lacking data in the Economist Democracy Index on Bermuda, Cape Verde and Palau no information about these countries (that make up 10% of the resettlement countries) can be provided.

22 8% resides in one of the countries with a non-defined regime type.

23 National Defense Authorization Act for Fiscal Year 2011, Pub. L. No. 111-383, 124 Stat. 4137 (2011), subtitle D sec. 1033(b).

24 It has, for example, been noted that the timing of Mujica’s decision to accept Guantanamo detainees coincided with a recent announcement of his nomination for the Nobel Peace Prize in 2014. Critics therefore consider this decision as a personally motivated strategic move to better his chances at winning the prize [55, 56].

25 Cable 09COLOMBO1012_a, The Maldives agrees to accept five Uyghur detainees, 4 November 2009, available at https://search.wikileaks.org/plusd/cables/09COLOMBO1012_a.html.

26 Cable 09SOFIA690_a, Bulgaria responds positively on Guantanamo, 7 December 2009, available at https://search.wikileaks.org/plusd/cables/09SOFIA690_a.html .

27 Cable 08STATE133238_a, Obtaining a commitment from the GOK to resettle the GTMO Uighurs, 20 December 2008, available at https://search.wikileaks.org/plusd/cables/08STATE133238_a.html.

28 It is important to note that other sources have stated that the negotiations regarding resettlement coincided with discussions on the renewal of a regular $ 200 million assistance package, but that there was no connection between the two [60]. Nonetheless, Palau ended up only accepting to resettle six detainees temporarily, until permanent homes for the six Uyghurs were found. Reportedly the country was compensated $600.000 for the temporary resettlement agreement [61].

29 Cable 09SOFIA690_a, Bulgaria responds positively on Guantanamo, 7 December 2009, available at https://search.wikileaks.org/plusd/cables/09SOFIA690_a.html.

30 Cable 09COLOMBO1012_a, The Maldives agrees to accept five Uyghur detainees, 4 November 2009, available at https://search.wikileaks.org/plusd/cables/09COLOMBO1012_a.html.

31 Cable 09ABUDHABI268_a, UAE asked again to consider hosting Guantanamo detainees, available at https://search.wikileaks.org/plusd/cables/09ABUDHABI268_a.html; Cable 09MUSCAT73_a, Oman unlikely to accept Guantanamo detainees.

32 Cable 09LJUBLJANA332_a, Slovenia: no easy solution for Guantanamo detainee resettlement, available at https://search.wikileaks.org/plusd/cables/09LJUBLJANA332_a.html; Cable 04PANAMA2362_a, Panama foreign minister meets ambassador Watt, available at https://search.wikileaks.org/plusd/cables/04PANAMA2362_a.html.

33 Bermuda is a British Overseas Territory, and thereby officially under the sovereignty of the UK.

34 EU Parliament adopted a joined resolution calling on its member states to accept detainees on February 4 2009, quickly after Obama announced his intentions to close the facility. See European Parliament resolution of 4 February 2009 on the return and resettlement of the Guantánamo detention facility inmates, P6_TA(2009)0045, available at https://www.europarl.europa.eu/sides/getDoc.do?pubRef=-//EP//NONSGML+TA+P6-TA-2009-0045+0+DOC+PDF+V0//EN.

35 The OSCE Office for Democratic Institutions and Human Rights strongly urged for the closure of the Guantanamo Bay facility and called upon OSCE member states to continue to resettle detainees on humanitarian grounds in a 2015 report [70].

36 For example, see https://www.hrw.org/sites/default/files/related_material/GTMO%20Resettlement%20QA.pdf

37 For example, see https://www.humanrightsfirst.org/sites/default/files/GTMO-Transfers-Issue-Brief.pdf

38 For example, see https://reprieve.org.uk/life-after-guantanamo/

39 For example, see https://www.cage.ngo/cage-africa-launches-global-petition-support-request-guantanamo-resettlement-sa

40 Cable 09LISBON399_a, Portugal requests two specific Guantanamo detainees, available at https://search.wikileaks.org/plusd/cables/09LISBON399_a.html.

41 Cable 09BERLIN1548_a, Germany renews consideration of Guantanamo detainees for resettlement, available at https://search.wikileaks.org/plusd/cables/09BERLIN1548_a.html; Cable 09HELSINKI312_a, Finland: Let’s talk detainees, just not Uighurs, available at https://search.wikileaks.org/plusd/cables/09HELSINKI312_a.html; Cable 09LISBON94_a , S/WCI Amb. Williamson discusses Guantanamo with Portugal, available at https://search.wikileaks.org/plusd/cables/09LISBON94_a.html; Cable 05PRAGUE1505_a, Czechs say no to the Uighurs, but agree to reconsider, available at https://search.wikileaks.org/plusd/cables/05PRAGUE1505_a.html; Cable 05COLOMBO1522_a, Request for resettlement assistance for certain detainees from Guantanamo: Sri Lanka, available at https://search.wikileaks.org/plusd/cables/05COLOMBO1522_a.html

42 Cable 09TIRANA722_a, Chinese visit Albania, available at https://search.wikileaks.org/plusd/cables/09TIRANA722_a.html.

43 For example, see BBC News [29], Blanchard [72] & CNN [73].

44 Summary of the Reengagement of Detainees Formely Held at Guantanamo Bay, Cuba, U.S. Director of National Intelligence, available at https://www.dni.gov/files/documents/Newsroom/Reports%20and%20Pubs/Final_Version_11-26-19_ATTACH_Declassified_Summary_Reengagement_of_GTMO_Detainees_19-1392_A-DNI_Approved.pd.pdf. Accessed 23 April 2020.

45 Translated from Spanish, original quote: *Usted no puede venir a una casa ajena con una cultura distinta a querer imponer su credo, como decía el Quijote ‘donde fueres haz como vieres’. Es elemental.*

46 Translated from Spanish, original quote: *La conducta de la gente que salió es pésima. De una absoluta falta de solidaridad con los que estaban allá. Porque si ellos hubieran cultivado otra imagen habrían facilitado que otros pudieran salir. Lo único que hicieron fue que tres o cuatro gobiernos de América Latina, que estaban por tomar una medida parecida, se retrajeran, ¿a quién perjudicaron?, a los otros presos de Guantánamo.*

47 Translated from Spanish, original quote: *Yo para venderle unos kilos de naranjas a Estados Unidos me tuve que bancar a cinco locos de Guantánamo.*

48 In late 2019 three Afghan ex-detainees were released form the Al-Razeen prison to the custody of Afghanistan, indicating that while not all detainees are still at the facility, the majority of detainees resettled to the UAE likely remain detained at Al-Razeen to this day [92].
